# Immune Cells as Mediators Between Gut Microbiota and Multiple Sclerosis: Insights From Mendelian Randomization

**DOI:** 10.1002/iid3.70475

**Published:** 2026-06-29

**Authors:** Pingping Ning, Xin Mu, Xiaohui Zhang, Yue Liu, Rui Yuan, Peng Tang, Rui Li

**Affiliations:** ^1^ Department of Geriatric Neurology Shaanxi Provincial People's Hospital Xi'an China; ^2^ Shaanxi Provincial Clinical Research Center for Geriatric Medicine Xi'an China; ^3^ Department of Neurology Chengdu First People's Hospital Chengdu China

**Keywords:** gut microbiota, immune cells, immune traits, mendelian randomization, multiple sclerosis

## Abstract

**Background:**

Multiple sclerosis (MS) is a neuroimmunological disorder marked by demyelination and neuronal damage, significantly impacting young individuals' health and quality of life. Recent studies suggest a pivotal role of the gut microbiota and immune system in MS development, though the precise mechanisms remain unclear.

**Methods:**

This study utilizes Mendelian randomization (MR) to explore the causal relationships between gut microbiota, immune traits, and MS. We conducted MR analysis using genetic data from 18,340 participants for gut microbiota, 3757 individuals for immune traits, and 47,429 MS cases alongside 68,374 controls.

**Results:**

Our results suggested six microbial taxa with nominally significant associations to MS, with two taxa (Roseburia and Ruminococcus2) exhibiting putative mediating trends through specific immune cell phenotypes (CD28 on CD28^+^ CD4^+^ T cells and CD45 on HLA‐DR^+^ CD8^+^ T cells). Mediation analysis demonstrated that 8.97% and 12.18% of the nominal effects of these taxa on MS were potentially mediated through the aforementioned immune traits, respectively. These results only represent exploratory trends and need to be interpreted with caution.

**Conclusion:**

These nominally significant exploratory findings provide hypothesis‐generating clues that immune cell activation, particularly in CD4^+^ and CD8^+^ T cells, might be involved in the microbiota‐MS relationship. These findings require validation in independent populations and experimental studies.

**Abbreviations**
AC: absolute cell
APCs: antigen‐presenting cells
CNS: central nervous system
DCs: dendritic cells
EBV: Epstein‐Barr virus
FDR: false discovery rate
GWAS: genome‐wide association studies
IMSGC: International Multiple Sclerosis Genetics Consortium
IVs: instrumental variables
IVW: inverse variance weighted
MFI: median fluorescence intensities
MP: morphological parameters
MR: mendelian randomization
MS: multiple sclerosis
RC: relative counts
SCFA: short‐chain fatty acid
SNPs: single nucleotide polymorphisms
TBNK: T cells, B cells, natural killer cells

## 1 Introduction

Multiple sclerosis (MS) is a neuroimmunological disorder of the central nervous system characterized by demyelination and neuronal damage, and is one of the most common causes of non‐traumatic disability in young individuals [1]. Recent global MS epidemiological data indicate that approximately 2.8 million people are affected by MS worldwide. Since 2013, the incidence of new MS cases has increased by 500,000 globally [2]. The incidence of MS in women is approximately three times higher than in men [3]. MS manifests with various phenotypes, with relapsing‐remitting MS being the most common. During relapse periods, patients may experience multiple acute neurological impairments, including motor weakness, sensory deficits, balance loss, vision deterioration, or blindness [4]. The long‐term accumulation of physical and cognitive disabilities in MS patients has significant social, economic, and personal impacts. In the United States, the annual economic burden of MS is approximately $85.4 billion [5]. The exact etiology of MS remains unclear. However, epidemiological and related studies suggest that interactions between environmental factors, lifestyle, and susceptibility genes may contribute to the onset of the disease [6]. Therefore, there is an urgent need to further elucidate its pathogenesis and identify biomarkers capable of effectively predicting and monitoring disease progression.

MS results from the combined effects of environmental factors, lifestyle, and genetic background. Known risk factors for MS include high latitude, female gender, smoking, adolescent obesity, low vitamin D levels caused by insufficient sunlight exposure and/or dietary intake, and Epstein‐Barr virus (EBV) infection, whereas the use of nicotine or alcohol, cytomegalovirus infection, and high caffeine intake are associated with a reduced risk of MS [7]. Moreover, recent Mendelian randomization (MR) studies have supported these associations [8, 9, 10]. Findings suggest that certain factors, such as smoking, EBV infection, and obesity, interact with HLA risk genes to influence adaptive immunity, thereby leading to autoimmune attacks on the nervous system [11, 12, 13]. Consequently, these environmental factors and lifestyle choices may influence the development of MS by altering immune traits. Therefore, we utilized MR to explore the potential relationship between immune traits and MS.

The intestine constitutes an integral component of the immune system and plays a pivotal role in immunomodulation. The gut microbiota serves as a critical immunoregulatory factor. Dysbiosis of the microbiota can lead to alterations in the production of microbial metabolites, which in turn may modify the activity of antigen‐presenting cells (APCs), T cells, and B cells, thereby contributing to the pathogenesis of autoimmune diseases [14]. An imbalance in the gut microbiota may elevate the risk of MS, although the precise mechanisms remain elusive. The influence of the gut microbiota on MS may not be direct, and immune cells are likely to play a crucial potential role in this process. However, there is a notable paucity of MR studies in this domain.

MR is a methodological approach that employs genetic variants as instrumental variables to investigate putative causal relationships between exposure factors and disease outcomes [15]. In contrast to conventional observational studies, MR leverages the principle of genetic randomization to effectively mitigate confounding biases and reverse causation, thereby offering more referential causal inferences. Initially, single nucleotide polymorphisms (SNPs) significantly associated with the exposure factor are identified through genome‐wide association studies (GWAS) or other genetic databases, and are subsequently filtered based on a predefined p‐value threshold to ensure a sufficiently strong association between the selected SNPs and the exposure factor. Furthermore, linkage disequilibrium analysis is applied to select SNPs located within regions of high linkage disequilibrium. Ultimately, these filtered SNPs are utilized as instrumental variables (IVs) for subsequent causal inference analyses. This causal inference process is analogous to a randomized controlled trial and provides more reliable clinical evidence compared to traditional correlational analyses [16].

**Figure 1:**
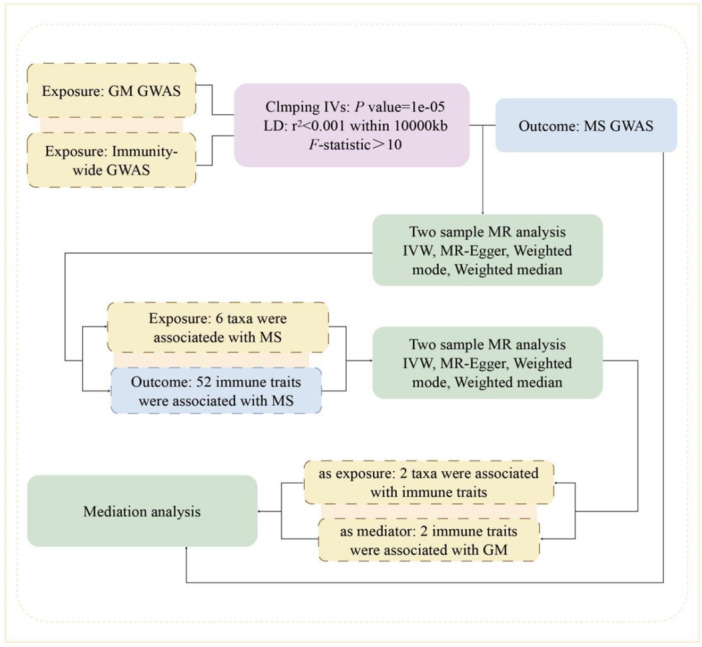
Flowchart of research design. GM, gut microbiota; GWAS, genome‐wide association studies; IVW, inverse variance weighted; MS, multiple sclerosis.

Building upon the aforementioned theoretical framework, we aim to employ the MR approach to investigate potential pathways linking gut microbiota, immune traits, and MS. Initially, we applied MR analysis to explore the potential relationship between gut microbiota and MS, identifying six gut microbial taxa with nominally significant associations to MS. Subsequently, we utilized MR analysis to examine the potential relationship between immune traits and MS, revealing 52 nominally significant immune traits associated with MS. Following this, we evaluated the potential relationships between these six gut microbial taxa and the 52 immune traits. Finally, we conducted a mediation analysis to identify potential intermediary immune traits linking gut microbiota and MS. The flowchart of the research design is shown in Figure 1.

## 2 Methods

### 2.1 Data Sources

The summary‐level data for gut microbiota were obtained from the MiBioGen consortium (https://mibiogen.gcc.rug.nl), comprising 18,340 participants from 24 cohorts, of which 78% were of European ancestry [17]. The MiBioGen consortium compiled and analyzed whole‐genome genotyping and 16S fecal microbiota data from participants. Only microbial taxa present in more than 10% of the samples were used to identify genetic loci influencing relative abundance, resulting in the identification of 211 taxa, including 131 genera, 35 families, 20 orders, 16 classes, and 9 phyla. The summary‐level data for 731 immune traits were derived from the GWAS summary statistics by Orru et al., which analyzed 3757 individuals from a Sardinian founder population using flow cytometry [18]. This dataset included 118 absolute cell (AC) counts, 389 median fluorescence intensities (MFI) reflecting surface antigen levels, 32 morphological parameters (MP), and 192 relative counts (RC) (ratios between cell populations). Specifically, it encompassed B cells, dendritic cells (DCs), T cells, monocytes, myeloid cells, T cells, B cells, natural killer cells (TBNK), and Treg panels at various maturation stages. The summary‐level data for MS were sourced from the International Multiple Sclerosis Genetics Consortium (IMSGC) GWAS summary statistics, which included 47,429 MS cases and 68,374 control subjects of European ancestry [19].

### 2.2 Data Extraction

The MR method used single nucleotide polymorphisms (SNPs) that were strongly associated with exposure factors as instrumental variables (IVs) to assess the putative causal effect of exposure on the outcome. We harmonized allele information of SNPs for exposure and outcome traits, and strictly excluded SNPs with mismatched alleles or uncertain strand information to maintain consistency in genetic effect directions. To qualify as IVs, three criteria must be met: (1) they must have a direct association with the exposure; (2) they must not be influenced by potential confounding factors; and (3) they must not be correlated with the outcome [20]. To meet the required standards, strict selection criteria were applied to the SNPs in this study. The exclusion criteria were as follows: (1) SNPs with a *p* value for association with the exposure ≥ 1 × 10^−5^; (2) weak instrumental variables with an F‐statistic < 10 [21]; (3) SNPs in linkage disequilibrium (LD) with an *r* ^2^ ≥ 0.001 and within a genetic distance of 10,000 kb [22]. (4) SNPs directly associated with multiple sclerosis; and (5) SNPs with genotyping errors or a sample missingness rate > 5%.

### 2.3 MR and Mediation Analysis

For polygenic MR analysis, the inverse variance weighted (IVW) method was adopted. While a fixed‐effects model was used initially, a random‐effects model was implemented upon detection of significant heterogeneity. If Cochran's Q statistic indicated significant heterogeneity (*p* < 0.05), the weighted median method was prioritized. Only gut microbiota and immune traits with no significant heterogeneity (het Q *p* > 0.05) and no significant pleiotropy (pleio *p* > 0.05) were included in the subsequent mediation analysis.

In the mediation analysis, if a gut microbiota and immune traits each have a nominal causal association with MS, and the gut microbiota also has a nominal causal association with the immune traits, a triangular relationship is formed. In this relationship, the gut microbiota serves as the exposure, immune traits as the mediator, and MS as the outcome. To ensure the biological plausibility of the associations between the gut microbiota and MS, the gut microbiota and immune traits, and immune traits and MS, only gut microbiota‐immune traits pairs that meet one of the following two criteria were included: (1) for gut microbiota positively associated with MS, the effect direction with the corresponding immune traits must align with the effect direction between immune traits and MS; (2) for gut microbiota negatively associated with MS, the effect direction with the related immune traits must be opposite to the effect direction of immune traits on MS. For the eligible gut microbiota and immune traits, mediation MR analysis was performed to identify potential relationships between the gut microbiota and immune traits that are putatively linked to MS. The univariate MR analysis was conducted by considering the selected gut microbiota as the exposure and the selected immune traits as the outcome. The formula used to determine the mediation effect proportion is: Mediation Proportion = βEM × βMO/βEO [23, 24], where βEM represents the MR putative causal effect of the exposure (E) on the mediator (M), βMO represents the MR putative causal effect of the mediator (M) on the outcome (O), and βEO represents the “total” effect of the exposure (E) on the outcome (O).

### 2.4 Sensitivity Analysis

For sensitivity analysis, Cochran's Q statistic calculated using the IVW method was employed to assess heterogeneity, with a *p* ＞ 0.05 indicating no significant heterogeneity [25]. If significant heterogeneity was detected, this was clearly indicated in the results along with the alternative analytical method employed. Findings with significant heterogeneity and no valid alternative method were excluded from subsequent analyses. In addition to Cochran's Q statistic, this study employed weighted median, MR Egger, and weighted mode methods as complementary sensitivity analyses. These approaches help verify the potential robustness of the causal effect estimates even when some instrumental variables may be invalid. The MR‐Egger intercept test was utilized to evaluate pleiotropy, with a *p* ＞ 0.05 indicating the absence of significant pleiotropic effects. Detailed results from each method are presented in Tables S1–S4. All MR analyses were conducted in R (version 4.3.2) using specific packages, including “TwoSampleMR (version 0.6.20)”, “tidyverse (version 2.0.2)”, “ggplot2 (version 3.5.1)”, “purrr (version 1.0.2)”, “data. table (version 1.16.2)”, “MendelR (version 9.2.38)” and “LDlinkR (version 1.4.0)”.

## 3 Results

### 3.1 Causal Relationship Between GM and MS

A total of 6 microbial taxa showed nominally significant associations with MS (uncorrected *p* < 0.05), whereas all associations became non‐significant after false discovery rate (FDR) correction (pFDR > 0.05). These findings should be interpreted as exploratory trends rather than statistically significant causal relationships.

**Figure 2:**
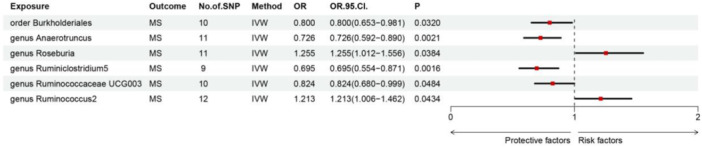
Genetic causality and correlation between gut microbiota and MS.

Univariate MR analysis revealed nominally significant associations between MS and six known microbial taxa. Among these, four taxa were negatively correlated with MS, while two taxa were positively correlated with MS (determined using the IVW method) (Figure 2). Heterogeneity analyses, the mean F‐statistic for each exposure factor, MR‐Egger intercept estimates and corresponding p‐values are detailed in Table S2. An intercept significantly deviating from zero indicates the presence of horizontal pleiotropy (*p* < 0.05), with its sign reflecting the direction of the pleiotropic effect. Detailed results of the multi‐method MR analysis for the six microbial taxa with nominal causal associations to MS are presented in Table S1. Among these, the genera *Anaerotruncus* and *Ruminiclostridium5* remained nominally significant in the weighted median method (OR 0.6732, *p* = 0.0049; OR 0.7153, *p* = 0.0335, respectively). For most taxa, the effect directions across MR‐Egger, weighted median, and weighted mode methods were consistent with the IVW results. However, notable discrepancies were observed for *Ruminococcaceae UCG003*: IVW suggested a protective effect (OR 0.84, *p* = 0.0484), while MR‐Egger showed a non‐significant effect in the opposite direction (OR 1.1585, *p* = 0.6723). Such discrepancies may arise due to low statistical power of MR‐Egger when the number of instrumental variables is small. Consistency between the weighted median method (robust to partially invalid IVs) and IVW supports the exploratory protective trend of this taxon. Among these microbial taxa, *Ruminiclostridium5* had the most significant nominal impact on MS risk (OR 0.695, 95% CI 0.554–0.871; *p* = 0.0016). We selected these six microbial taxa for further mediation MR analysis as an exploratory study.

### 3.2 Causal Relationship Between Immune Traits and MS

A total of 52 immune traits showed nominally significant associations with MS (*p* < 0.05), and all associations were non‐significant after FDR correction (pFDR > 0.05). These results only represent exploratory trends for the immune trait‐MS relationship.

**Figure 3:**
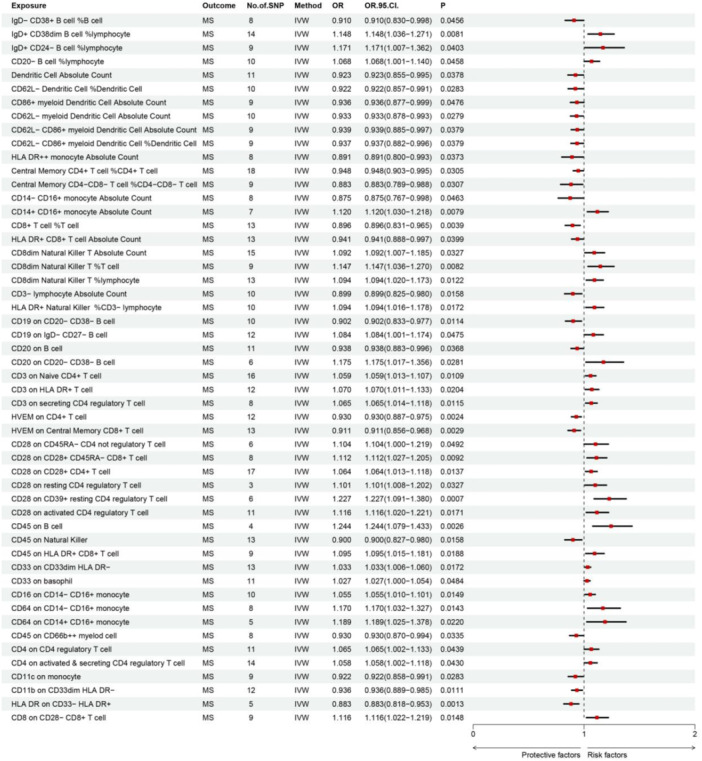
Genetic causality and correlation between immune traits and MS.

A total of 52 immune traits were found to be nominally associated with MS (Figure 3). Heterogeneity analyses, the mean F‐statistic for each exposure factor, MR‐Egger intercept estimates, and corresponding p‐values are detailed in Table S4. An intercept significantly deviating from zero indicates the presence of horizontal pleiotropy (*p* < 0.05), with its sign reflecting the direction of the pleiotropic effect. Among the 52 immune traits with nominal causal associations to MS in the multi‐method MR analysis, results from the MR‐Egger, weighted median, and weighted mode methods were generally consistent in direction with those from the IVW method for the majority of traits. Detailed results are presented in Table S3. All results underwent comprehensive assessment for heterogeneity and pleiotropy. Subsequently, these 52 immune traits were selected for further mediation MR analysis as an exploratory study.

### 3.3 Mediation Analysis Results

**Figure 4:**
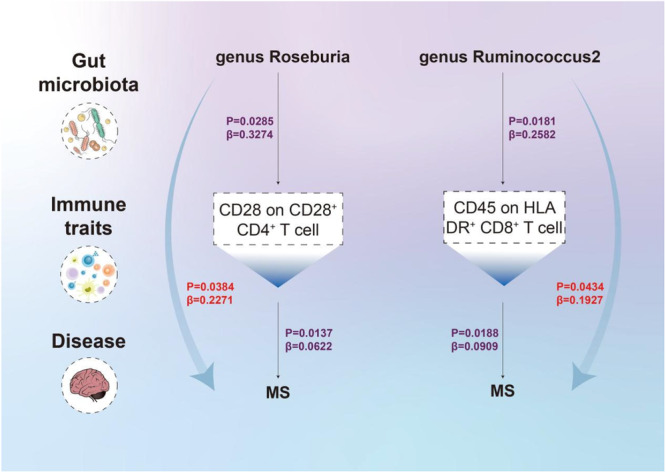
Mediation analysis results. MS, multiple sclerosis.

To explore the potential mediating relationship between the gut microbiota and immune traits, mediation MR analysis was performed. We selected 6 microbial taxa and 52 immune traits phenotypes for MR analysis, which revealed potential causal relationships between 2 microbial taxa and 2 immune cell phenotypes. These microbial taxa and immune cell phenotypes were selected as exposure and mediator factors for the mediation analysis. All results were nominally significant and need to be interpreted with caution. The proportion of mediation effects was calculated using the following formula: Mediation ratio = Indirect effect/Direct effect, where the indirect effect = βEM × βMO and the direct effect = βEO. The indirect effect's 95% confidence interval (CI) was computed using the produlin method, with a non‐zero CI indicating potential referential value. Furthermore, by dividing the indirect effect's 95% CI by the direct effect, we obtained the 95% CI for the mediation effect. The results are presented in Figure 4 and Table 1.

**Table 1:** Mediation analysis results.

Exposure	Mediator	Direct effect (βEM ± SE)	Direct effect (βMO ± SE)	Direct effect (βEO ± SE)	Indirect effect (βEM × βMO ± SE)	95%CI	Proportion mediated (βEM × βMO/βEO)
Genus Roseburia	CD28 on CD28^+^ CD4^+^ T cell	0.3274 ± 0.1495	0.0622 ± 0.0252	0.2271 ± 0.1096	0.0204 ± 0.0130	0.0005–0.0504	8.97% (0.21%–22.19%)
Genus Ruminococcus2	CD45 on HLA DR^+^ CD8^+^ T cell	0.2582 ± 0.1092	0.0909 ± 0.0387	0.1927 ± 0.0954	0.0235 ± 0.0147	0.0010–0.0574	12.18% (0.48%–29.79%)

*Note:* βEM represents the MR causal effect of exposure E on mediator M, βMO denotes the MR causal effect of mediator M on outcome O, and βEO indicates the ‘total’ effect of exposure E on outcome O. The 95% CI refers to the 95% confidence interval for the indirect effect.

Our exploratory analysis suggests that 8.97% of the effect of genus *Roseburia* on MS is potentially mediated via CD28 on CD28^+^ CD4^+^ T cells, with the mediation effect's 95% CI greater than 0. Similarly, 12.18% of the effect of genus *Ruminococcus2* on MS is potentially mediated via CD45 on HLA DR^+^ CD8^+^ T cells, with the mediation effect's 95% CI also greater than 0. These findings only represent exploratory trends, suggesting that CD28 on CD28^+^ CD4^+^ T cells and CD45 on HLA DR^+^ CD8^+^ T cells may serve as potential mediator factors in the interaction between the gut microbiota and MS.

## 4 Discussion

Interpretation caution: All associations reported in this study were only nominally significant (uncorrected *p* < 0.05) and became non‐significant after false discovery rate (FDR) correction. Therefore, none of the findings should be interpreted as statistically significant causal relationships. The results are exploratory and hypothesis‐generating only, serving to provide tentative clues for future research rather than definitive conclusions.

This study employs the MR approach to investigate the putative causal relationships between gut microbiota, immune traits, and MS, generating two exploratory hypotheses that warrant further investigation. Firstly, the genus *Rosebur*ia may be involved in the onset of MS by affecting the expression of CD28 on CD28 + CD4 + T cells. Secondly, the genus *Ruminococcus2* may be involved in the progression of MS by modulating the expression of CD45 on HLA‐DR + CD8 + T cells. The results of this study only provide exploratory clues for potential causal associations, not only highlighting the potential putative causal link between gut microbiota and MS but also identifying the potential mediating role of immune cells in this relationship, offering novel hypothesis directions for the pathogenesis of MS.

If the observed nominal associations were to be confirmed in future studies, one potential mechanistic explanation could involve short‐chain fatty acids (SCFAs). Both genus *Roseburia* and *Ruminococcus2* belong to the same group of short‐chain fatty acid (SCFA)‐producing bacteria. SCFAs are the primary products of bacterial fermentation of fiber, including acetate, propionate, and butyrate. These metabolites provide energy to colonic epithelial cells and regulate inflammatory responses [26]. SCFAs play a crucial role in the interactions between the microbiota, gut, and brain [27]. Evidence suggests that the gut bacterial composition and SCFA levels are altered in MS patients, with a reduction in SCFAs due to changes in the gut microbiota. The decrease in SCFAs leads to impaired Treg cell function. Additionally, oral administration of propionate has been shown to exert neuroprotective effects, such as increased availability of propionate in cerebrospinal fluid, which improves clinical outcomes and alters deep gray matter composition [28]. Another study demonstrated that treatment with butyrate ester in an MS mouse model effectively alleviates clinical symptoms and improves the histopathological features of the central nervous system. This study also observed a reduction in effector T cells, including Th1 and Th17 cells, in the central nervous system and gut lamina propria of the butyrate ester treatment group, while the proportion of regulatory T cells and IL‐10 secretion in peripheral immune organs increased [29].

However, some studies suggest that under certain conditions, SCFAs may exert a pro‐inflammatory effect, which is largely dependent on factors such as the type and concentration of SCFAs and the composition of the gut microbiota [30]. For example, butyrate at low concentrations can effectively promote the generation of regulatory T cells, but excessive concentrations may lead to the overactivation of certain immune cells [31]. Some studies have found that high concentrations of SCFAs may exacerbate T cell activation, particularly Th17 cells, which are key pro‐inflammatory factors in MS. IL‐17 produced by Th17 cells is closely associated with the inflammatory response in MS. Furthermore, SCFAs at specific concentrations may enhance the secretion of pro‐inflammatory cytokines, such as IL‐6 and TNF‐α, thereby exacerbating immune responses and inflammation.

Existing studies suggest that there may be a notable correlation between the gut microbiota and the expression of CD28 on CD28^+^ CD4^+^ T cells as well as CD45 on HLA‐DR^+^ CD8^+^ T cells. Case‐control studies have indicated a positive correlation between certain gut microbiota and CD4^+^ T cells and CD8^+^ T cells [32, 33].

In this study, CD28 on CD28^+^ CD4^+^ T cells was identified as a potential risk factor for MS, with gut microbiota potentially promoting the onset of MS by increasing the levels of CD28 on CD28^+^ CD4^+^ T cells. This finding is consistent with previous research, which has shown that the expression of CD28 on CD4^+^ T cells is associated with an increased risk of MS [34]. CD28 is a co‐stimulatory receptor on the surface of T cells that binds to co‐stimulatory molecules CD80 and CD86, promoting T cell activation and proliferation. When T cells recognize antigens through the T cell receptor (TCR), the binding of CD28 to its ligands enhances T cell activation signals, thereby triggering downstream immune responses [35]. In MS patients, the activation of CD28^+^ CD4^+^ T cells is typically enhanced through this co‐stimulatory mechanism [36]. CD28^+^ T cells are not only one of the pathogenic T cell subsets in MS but also participate in the regulation of demyelination [37]. Studies have shown that CD28^+^ CD4^+^ T cells in MS patients may be aberrantly activated in the context of immune tolerance dysregulation. Under normal circumstances, the activation of CD28 helps maintain immune system balance, but in MS patients, excessive CD28 signaling may lead to T cells mounting immune responses against neuroantigens, thereby attacking myelin [38]. Over‐activated CD28^+^ CD4^+^ T cells secrete cytokines such as interferon‐γ and tumor necrosis factor‐α, and induce other immune cells, such as B cells and macrophages, to participate in immune responses, thereby exacerbating inflammation and damage to the nervous system. Moreover, the expression level of CD28 and the activation state of T cells may be closely related to the clinical manifestations and disease activity of MS. For instance, high levels of CD28^+^ CD4^+^ T cells may indicate increased disease activity, particularly during acute flare‐ups, where excessive cytokine secretion may lead to further neuronal damage. Therefore, CD28‐targeting inhibitors may represent a new therapeutic strategy for MS. By reducing the over‐activation of CD28^+^ T cells, it may help suppress immune‐mediated damage and slow disease progression [34].

Although the primary pathological features of MS are typically associated with the CD4^+^ T cell subsets (Th1 and Th17 cells), CD8^+^ T cells may also play a crucial role in the disease process, particularly in myelin destruction and neurodegeneration [39]. In MS patients, CD8^+^ T cells can be activated through the HLA‐DR pathway and initiate immune attacks against neural antigens such as myelin. The expression of HLA‐DR indicates that these CD8^+^ T cells are in an activated state, potentially enhancing their responsiveness to central nervous system (CNS)‐specific antigens, thereby contributing to demyelination and neurological dysfunction. Moreover, studies have reported a slightly elevated number of activated CD8^+^ T cells in MS patients [40]. Another study evaluating the role of T cells in MS demonstrated that myelin‐specific CD8 + T cells are involved in disease pathogenesis [41]. In MS, the dysregulation of immune tolerance mechanisms leads to the aberrant activation of T cells that would otherwise remain tolerant to CNS antigens. CD8^+^ T cells may be erroneously activated in this process, attacking neurons or myelin. These HLA‐DR^+^ CD8^+^ T cells not only recognize CNS‐specific antigens but may also may exert cytotoxic effects and release pro‐inflammatory cytokines, thereby causing further neuronal damage [39]. Given the close association between the activation state of these cells and the immune response in MS, assessing the levels or activation status of CD45^+^ HLA‐DR^+^ CD8^+^ T cells may provide valuable insights into disease activity and the intensity of the immune response.

Our exploratory data suggest that CD45^+^ HLA‐DR^+^ CD8^+^ T cells may serve as a potential risk factor for MS, potentially increasing the incidence of the disease through the activation of additional mechanisms. Therefore, further studies are necessary to elucidate the potential role of CD45^+^ HLA‐DR^+^ CD8^+^ T cells in the pathogenesis of MS. Moreover, the activation state of these cells may serves as a potential marker of disease activity in MS, and future therapeutic strategies targeting these cells may help to slow or control disease progression.

Our study demonstrates that MR analysis can identify certain gut microbiota and immune traits potentially associated with MS. By combining two‐stage MR analysis and mediation analysis, we successfully linked gut microbiota with immune traits and constructed a hypothetical pathway through which the gut microbiota may influences MS via immune traits. CD28 on CD28^+^ CD4^+^ T cells and CD45^+^ HLA‐DR^+^ CD8^+^ T cells were identified as potential mediating immune traits. Despite employing rigorous MR methodology, there are still some limitations in this study.

First, the selection of gut microbiota relied on GWAS data (MiBioGen) with inherent limitations of weak genetic instruments and low explained variance, a common issue in microbiome‐based MR studies. Although all F‐statistics > 10 avoided weak instrument bias, the low heritability of microbial taxa limited the statistical power of causal inference, and this is also a key reason for non‐significant results after FDR correction. Additionally, GWAS data may not fully capture all MS‐associated microbiota characteristics, leading to potential under‐inclusion of relevant taxa. Second, the immune traits data were derived from the founder population of Sardinia, which may not fully represent immune traits distribution patterns in other populations. Further cross‐population studies will be beneficial for validating these findings. Third, although MR analysis reduces confounding bias through genetic instrumental variables, genetic variants may not fully reflect the biological mechanisms of the exposures. Fourth, certain microbial taxa and immune traits showed directional result inconsistencies between the MR‐Egger method and other MR approaches, which may stem from the low statistical power of MR‐Egger for exposures with few instrumental variables, weak genetic heritability of the measured traits, and unmeasured confounding in GWAS data, leading to compromised stability of effect estimates for individual taxa/traits. Fifth, MR‐PRESSO analysis was not performed due to the high computational burden of 58 exposure factors, which may lead to unrecognized abnormal SNPs and unstable causal effect estimates; reverse MR analysis could not be conducted for lack of valid instrumental variables, resulting in untested causal direction of all nominally significant associations and thus an inability to rule out reverse causality; multivariable MR and colocalization analyses were not implemented, as genetic instrument overlap complicates multivariable modeling and nominal associations lack sufficient statistical power for reliable colocalization, which limits the adjustment of confounding factors between exposures and the verification of shared genetic signals for gut microbiota/immune traits and MS. These analyses should be prioritized in future well‐powered studies to validate the proposed microbiota‐immune‐MS axis. Furthermore, potential reasons for the absence of significant findings after FDR correction include: (1) the limited genetic heritability explained by the gut microbiota and immune traits included in this study; (2) insufficient statistical power due to the sample size, given that the study population was predominantly of European descent; and 3) the fact that gut microbiota are regulated by multiple factors such as diet and environment, making it challenging to fully capture their complex relationship with MS using genetic data alone. Therefore, additional experimental validation is needed to confirm the biological plausibility of these causal inferences.

Our study exploratorily characterizes potential regulatory trends among gut microbiota, immune traits, and MS, suggesting a hypothetical framework (gut microbiota‐immune traits‐MS axis) that may warrant further investigation. These findings should be considered hypothesis‐generating rather than conclusive. Additionally, CD28 on CD28 + CD4 + T cells and CD45 on HLA‐DR + CD8 + T cells are identified as potential mediating immune traits in the microbiota‐MS crosstalk, which provides exploratory insights into MS pathogenesis. Notably, our results are only nominally significant and non‐significant after FDR correction, thus requiring further validation via large‐scale cross‐population MR analyses and in vitro/in vivo functional experiments.

## Author Contributions

**Pingping Ning:** conceptualization, methodology, software, validation, investigation, formal analysis, funding acquisition, visualization, writing – original draft, writing – review and editing. **Xin Mu:** conceptualization, methodology, software, investigation, validation, formal analysis, writing – original draft, writing – review and editing. **Xiaohui Zhang:** methodology, software, data curation, investigation. **Yue Liu:** methodology, software, data curation, investigation. **Rui Yuan:** methodology, software, data curation, investigation. **Peng Tang:** methodology, software, data curation, investigation. **Rui Li:** conceptualization, supervision, project administration, writing – review and editing.

## Ethics Statement

The authors have nothing to report.

## Conflicts of Interest

The authors declare no conflicts of interest.

## Supporting information

Supporting File 1:

Supporting File 2:

## Data Availability

The data that support the findings of this study are openly available in ieu OpenGWAS project at https://gwas.mrcieu.ac.uk/.
